# Graphene Oxide Nanosheets Reduce Astrocyte Reactivity to Inflammation and Ameliorate Experimental Autoimmune Encephalomyelitis

**DOI:** 10.1021/acsnano.2c06609

**Published:** 2023-01-24

**Authors:** Giuseppe Di Mauro, Roberta Amoriello, Neus Lozano, Alberto Carnasciali, Daniele Guasti, Maurizio Becucci, Giada Cellot, Kostas Kostarelos, Clara Ballerini, Laura Ballerini

**Affiliations:** † International School for Advanced Studies (SISSA/ISAS), 34136Trieste, Italy; ‡ Dipartimento di Medicina Sperimentale e Clinica, University of Florence, 50139Florence, Italy; § Catalan Institute of Nanoscience and Nanotechnology (ICN2), 08193Barcelona, Spain; ⊥ Dipartimento di Chimica “Ugo Schiff”, DICUS, University of Florence, 50139Florence, Italy; ¶ Nanomedicine Lab, and Faculty of Biology, Medicine & Health, The National Graphene Institute, University of Manchester, ManchesterM13 9PL, United Kingdom

**Keywords:** hemichannels, reactive astrocytes, graphene oxide, drug delivery, nanomedicine

## Abstract

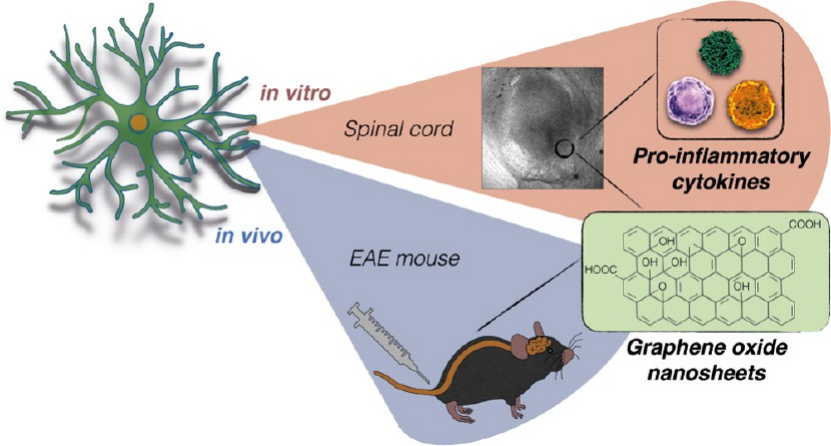

In neuroinflammation, astrocytes play multifaceted roles that regulate the neuronal environment. Astrocytes sense and respond to pro-inflammatory cytokines (CKs) and, by a repertoire of intracellular Ca^2+^ signaling, contribute to disease progression. Therapeutic approaches wish to reduce the overactivation in Ca^2+^ signaling in inflammatory-reactive astrocytes to restore dysregulated cellular changes. Cell-targeting therapeutics might take advantage by the use of nanomaterial-multifunctional platforms such as graphene oxide (GO). GO biomedical applications in the nervous system involve therapeutic delivery and sensing, and GO flakes were shown to enable interfacing of neuronal and glial membrane dynamics. We exploit organotypic spinal cord cultures and optical imaging to explore Ca^2+^ changes in astrocytes, and we report, when spinal tissue is exposed to CKs, neuroinflammatory-associated modulation of resident glia. We show the efficacy of GO to revert these dynamic changes in astrocytic reactivity to CKs, and we translate this potential in an animal model of immune-mediated neuroinflammatory disease.

Negative and maladaptive features of neuroinflammation contribute to the initiation and development of diverse diseases of the central nervous system (CNS),^1^ which include chronic neurodegenerative pathologies such as multiple sclerosis, Alzheimer’s and Parkinson’s diseases,^2−4^ or acute injuries such as stroke and traumatic injury.^5,6^ CNS inflammation is fueled by the production of mediators, ultimately released by CNS resident glia (microglia and astrocytes), endothelial cells, and peripherally derived immune cells. Astrocytes, the key regulators of CNS homeostasis,^7^ are active players in neuroinflammation, and recent research has shown that these CNS cells, by connexin hemichannel (HC) increased permeability, release pro-inflammatory factors and may contribute to CNS damage progression.^8,9^ Gaining insight into the cellular mechanisms that sustain neuroinflammation spreading is instrumental to development of future therapeutic tools, in particular to target core mediators common to a vast range of CNS pathologies.^1,10^

Graphene-based nanotechnology approaches are increasingly attracting the attention of biomedical scientists^11,12^ and can offer innovative solutions in neurology.^13,14^ In this context, graphene oxide (GO) is emerging as a potential multifunctional platform for therapeutic neuromodulation.^15−17^ We have previously reported the ability of thin, medical grade, and endotoxin-free GO nanosheets with small (100–400 nm) lateral dimensions (s-GO) to selectively target CNS subcellular components, such as synapses^18,19^ or modulate astrocyte membrane dynamics.^20^ More recently, we exploited the potential application of s-GO by targeting *in vivo* dysfunctional brain excitatory synapses and preventing the development of anxiety disorders in rats.^21^ Despite such promising advances in s-GO control over synaptic activity, relatively fewer studies investigated the interaction of graphene-based materials, in particular s-GO, with CNS glial cells,^22^ and it has not been addressed whether s-GO could interfere with astrocyte reactivity during neuroinflammation.

Here, we explore, by live imaging astrocyte Ca^2+^ signal dynamics in spinal-slice cultures, an in vitro system where the sensory-motor cytoarchitecture is preserved in 3D tissue retaining spinal cord resident cells.^23,24^ Incubation (6 h) with a pro-inflammatory cytokines (CKs) cocktail (TNF-α, IL-1β and GM-CSF^25−29^) ignited aberrant Ca^2+^ signaling in astrocytes, accompanied by morphological changes in reactive resident glia, altered synaptic activity, and increased connexin HC permeability. These functional markers of inflammation^25,26^ were all effectively down-regulated by s-GO (6 h) incubation, and s-GO specifically impaired HC permeability when pharmacologically increased. Last, we attempted to translate this protective effect of s-GO in a mouse model of experimental autoimmune encephalitis (EAE^30,31^), one of the most reliable rodent models for multiple sclerosis where, upon repeated intravenous delivery, we report that s-GO preserved its ability to reduce spinal astrogliosis and control neuronal cell loss in vivo. More interestingly, such histological changes were associated with an overall improved clinical score.

## Results and Discussion

### Graphene Oxide Synthesis and Characterization

**Figure 1:**
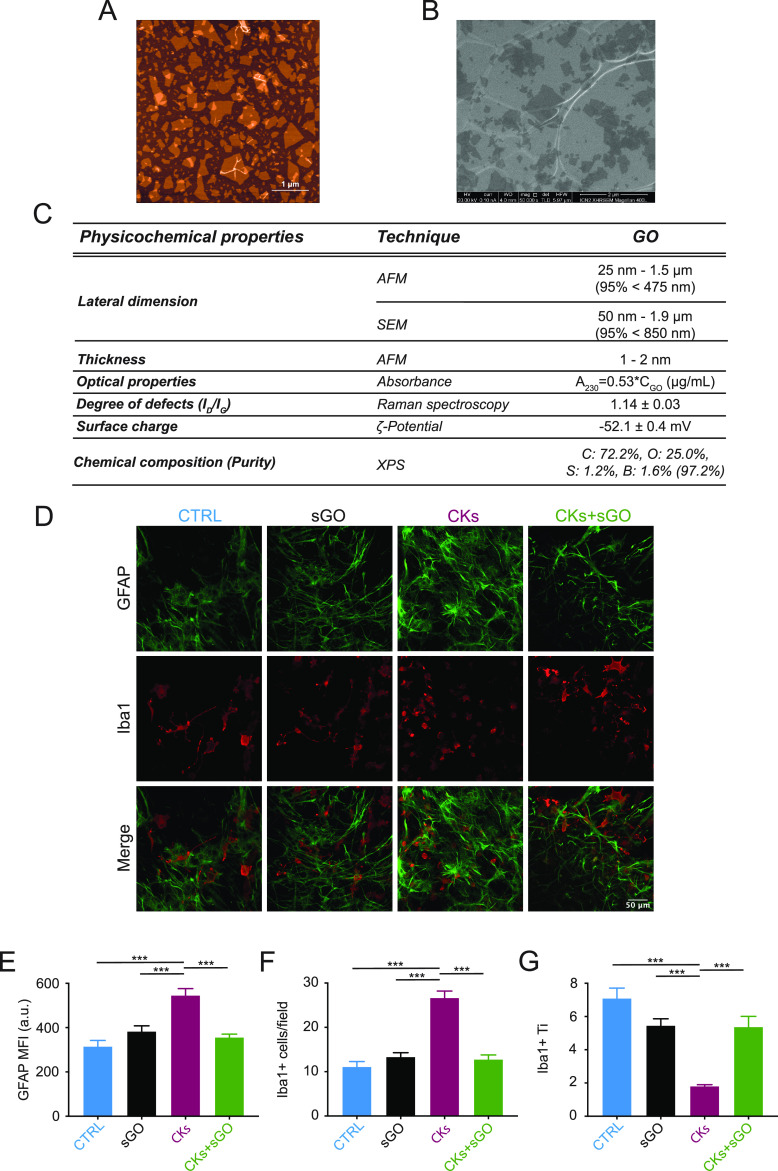
s-GO characterization and their impact on the morphology of spinal neuroglia exposed to pro-inflammatory CKs. Characterization of s-GO nanosheets. (A) Height AFM image. (B) SEM micrograph. (C) Summary of physicochemical properties by different analytical, spectroscopic, and microscopic techniques. (D) Representative confocal micrographs of ventral areas in spinal cultures with astrocytes and microglia visualized using anti-GFAP (in green) and anti-Iba1 (in red) antibodies. Bar plots summarize (E) GFAP fluorescence intensity, (F) Iba1 positive cell density, and (G) Ti; *** *P* < 0.001.

The s-GO material used in these studies has been synthesized according to the protocols that have already been reported and characterized extensively in our previous published work.^18,19,32,33^ The specific batch of GO nanosheets used here was thin (1–3 layers) characterized by lateral dimensions of 100–400 nm, with reproducibly purity and well demonstrated biotolerability. Figure 1A–C describe in detail the physicochemical characterization of the batch of GO nanoflakes used in these studies.

Our previous results in vitro and in vivo strongly support the use of s-GO with this specific range of dimensions in complex biological environments,^18,19,21,33,34^ and in particular due to s-GO lack of in vitro and in vivo cytotoxic responses.^33,35−37^

### Glial Cell Reactivity Due to Inflammatory Danger Is Controlled by Graphene Oxide

We used organotypic spinal cord cultures to monitor resident astrocytes and microglia reactivity to neuroinflammation and to explore s-GO impact on activated neuroglia. Spinal explant cultures are a convenient *in vitro* model to reproduce an inflammatory environment and to gain insight into CNS responses to danger signals.^25−27^ To activate resident glial cells, we adopted a well-characterized neuroinflammation paradigm where organotypic cultures (2 weeks *in vitro*, WIV) were incubated (6 h) with a mixture of pro-inflammatory CKs (TNF-α, IL-1β, GM-CSF, 10 ng/mL) reported to be released in EAE and to induce CNS reactivity.^25−29^ We (co)incubated separate groups of untreated or CK-treated cultures with s-GO (6 h) at a concentration of 25 μg/mL.^18,19,21,33^ In all four culture groups we used the specific markers, glial fibrillary acidic protein (GFAP) and ionized calcium-binding adapter molecule 1 (Iba1), to monitor by immunofluorescence microscopy the morphology of astrocytes and microglia, respectively (Figure 1D).

CKs induced a massive enhancement in GFAP mean fluorescence intensity (MFI; from 315 ± 27 au in control to 545 ± 30 au in CKs; n = 9 fields each condition; Figure 1D,E) and significantly increased Iba1-microglia cell density (11 ± 1 cells/field control and 26 ± 1 cells/field CKs, *P* < 0.001; Figure 1D,F) with a typical reduction in Iba1-positive cell transformation index (Ti; 7.09 ± 0.61 control and 1.81 ± 0.08 CKs, *P* < 0.001; Figure 1G), supportive of CKs-induced active (ameboid) microglia.^38−40^ All these changes are in accordance with astrocyte and microglia activation upon inflammation in spinal explant cultures.^25,26^ s-GO incubation per se did not alter glia morphology in terms of GFAP MFI (383 ± 25 au in n = 9), Iba1-positive cell density (13 ± 1 cells/field), and Ti (5.45 ± 0.41, *P* > 0.033 control vs s-GO; Figure 1C,E,F). When coapplied to CKs, s-GO prevented the increase in MFI of GFAP (356 ± 14 au, n = 9 fields) as well as Iba1-positive microglia changes in cell density (12 ± 1 cells/field) and Ti (5.38 ± 0.62; Figure 1C–F; *P* < 0.001 CKs vs CKs+s-GO). These results suggested that s-GO nanosheets exhibited a modulatory action on the development of neuroinflammation in spinal cord slices.

### s-GO Protect Spinal Astrocytes and Neurons from Dysfunctional Signaling Triggered by CKs

**Figure 2:**
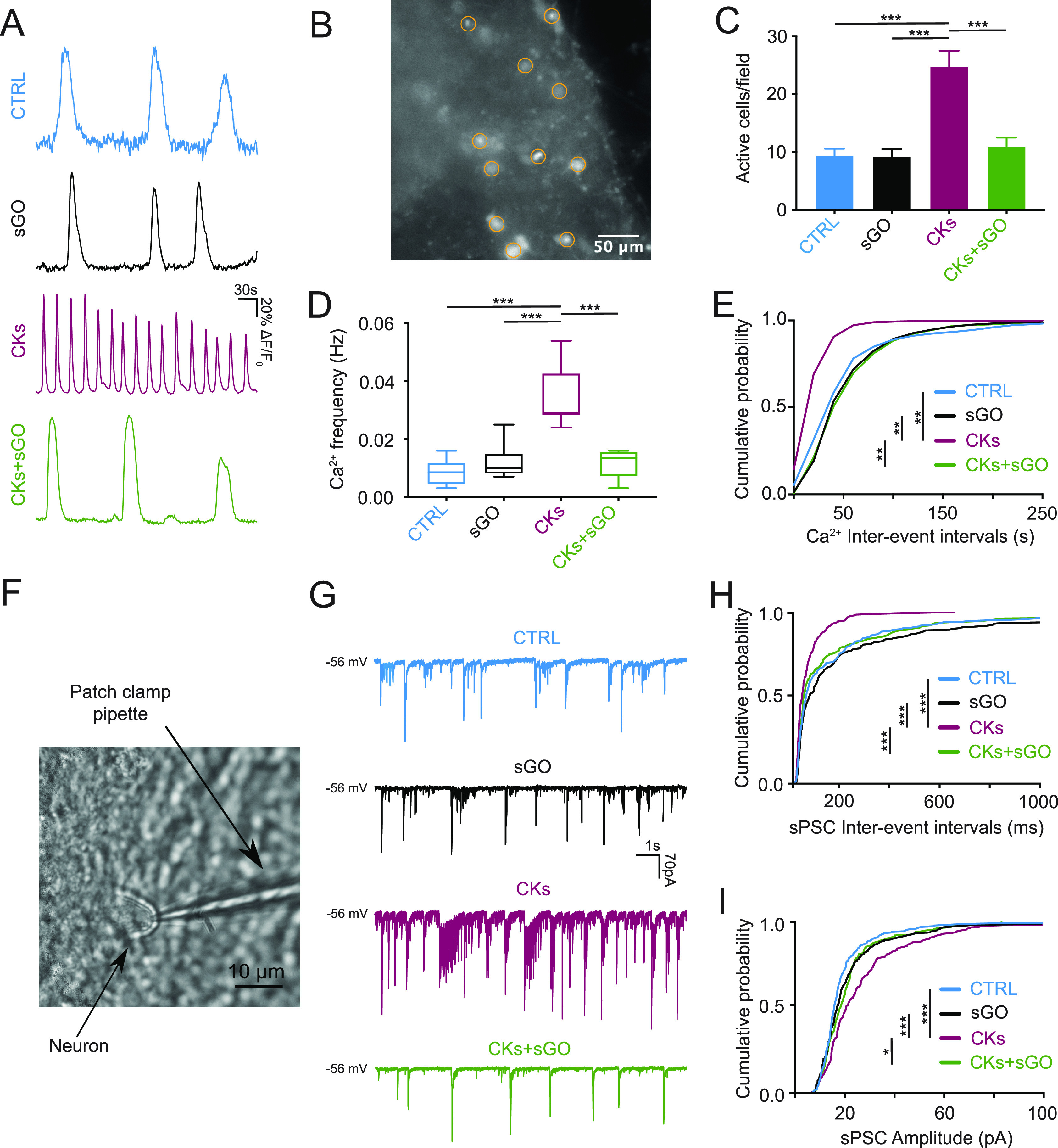
sGO prevent the emergence of CKs-induced dysfunctional Ca2+ signaling in astrocytes and altered synaptic signaling in neurons. (A) Representative fluorescent traces depicting astrocytes calcium activity in control, sGO, CKs, and CKs+sGO conditions. (B) Snapshot of the ventral area visualized by Fluo-4 AM, ROIs highlight active cells (control culture). (C) Bar plot of active astrocytes, (D) box-plot of calcium events frequency, and (E) cumulative distribution of their inter-event intervals. (F) Bright field image of a ventral interneuron targeted by a patch pipette. (G) Representative voltage clamp tracings in control, sGO, CKs, and CKs+sGO conditions, and cumulative probability plots of PSCs (H) inter-event intervals and (I) amplitudes. * *P* < 0.033, ** *P* < 0.002, *** *P* < 0.001.

Astrocytes, via complex spatial-temporal changes in cytoplasmic Ca^2+^ concentrations, integrate and propagate signals in the CNS to respond to a variety of external stimuli.^41,42^ Slow calcium oscillations, generated by astrocytes in the spinal cord,^43,44^ were isolated and measured in the ventral horn of organotypic cultures (2 WIV) during calcium imaging experiments with the calcium dye Fluo-4 AM, and in the continuous presence of tetrodotoxin (TTX, 1 μM; see Experimental Section) a sodium fast-inactivating blocker that abolished action potentials and removed back-ground neuronal activity.^25,45^Figure 2A shows fluorescent tracings depicting intracellular calcium dynamics in astrocytes recorded from the sampled area (320 × 320 μm^2^, Figure 2B) of the ventral spinal horn in control and CKs cultures, with or without s-GO. In control glial population, calcium dynamics emerge as slow oscillations occurring at a low pace. When in the presence of s-GO, the number of active cells and the frequency of calcium oscillations did not change (Figure 2C–E). Resident glial cell reactivity to pro-inflammatory CKs more than doubled the amounts of active astrocytes (from 9 ± 1 cells/field in n = 12 control to 24 ± 2 cells/field in n = 10 CKs, *P* < 0.001, Figure 2B,C) and strongly increased events frequency (from 0.008 ± 0.001 Hz in control to 0.034 ± 0.003 Hz in CKs, *P* < 0.001, Figure 2A–D). s-GO, when coapplied to CKs, efficiently prevented the boost in astrocyte calcium oscillations, leaving a basal activity reminiscent of control (11 ± 1 cells/field and 0.011 ± 0.001 Hz, n = 8, *P* < 0.001; Figure 2A–D). In Figure 2E, the changes in calcium dynamics are summarized as inter-event intervals and compared among the culture groups as cumulative probability plots (control, s-GO and CKs+s-GO vs CKs *P* < 0.002; s-GO, and CKs+s-GO vs control *P* > 0.033).

In organotypic cultures, astrocyte aberrant calcium signals contribute to cytokines and chemokines release due to inflammation.^25^ CKs directly or via resident cells boost network excitability resulting in an enhanced occurrence of spontaneous postsynaptic currents (sPSC^26,27^).

Patch clamped ventral interneurons (Figure 2F) displayed an intense basal synaptic activity in control,^24,46^ and representative tracings in Figure 2G show the occurrence of heterogeneous sPSCs, which were not affected by s-GO (sPSC inter event intervals from 188 ± 26 ms in controls n = 9 to 236 ± 36 ms in s-GO n = 8; sPCS amplitude from 19 ± 1pA in control to 21 ± 1pA in s-GO Figure 2G–I). Conversely, CKs treatment induced a strong increase in synaptic activity, completely prevented by s-GO coincubation (sPSC inter event intervals from 55 ± 5 ms in CKs n = 8 to 166 ± 22 ms in CKs+s-GO n = 7; sPCS amplitude from 27 ± 1 pA in CKs to 21 ± 1 pA in CKs+s-GO; cumulative distribution in Figure 2H, control, s-GO and CKS+s-GO vs CKs < 0.001, in Figure 2I control, s-GO vs CKs *P* < 0.001, CKs vs CKs+s-GO *P* < 0.033).

The protective effects of s-GO on CKs-mediated overactivation of calcium signaling in glial cells, and of synaptic signaling in neurons, strengthen the hypothesis of s-GO modulatory action over neuroinflammation. Such a modulatory role is also supported by s-GO ability to control the production and release of cytokines (IL-1β; IL-6; TNF-α) and chemokine (CCL2; CXCL10) measured by Milliplex assay of the supernatants harvested from organotypic slices^25^ in CKs or CK+s-GO treated slices (Figure S1).

### s-GO Treatment Prevents CKs-Induced Increase in Glial Hemichannels Opening

**Figure 3:**
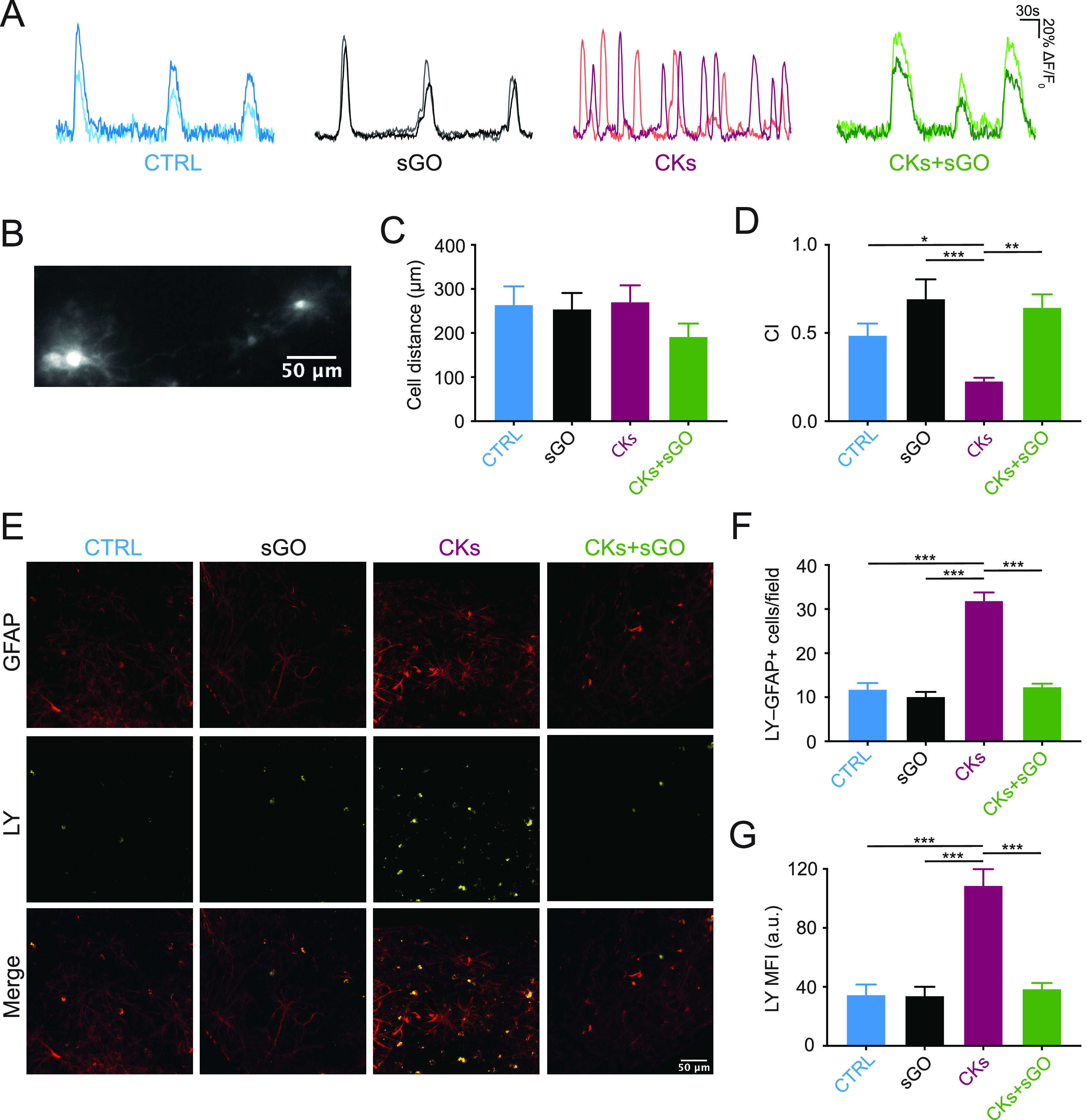
s-GO modulate spinal astrocytes HCs activation by pro-inflammatory CKs. (A) Superimposed fluorescent traces of Ca^2+^ oscillations in synchronized or unsynchronized pairs of astrocytes recorded in control, s-GO, CKs, and CKs+s-GO conditions. (B) Snapshot of astrocytes pair in Fluo-4 AM. Bar plots summarize the (C) mean astrocyte pair distance and (D) CI. (E) Confocal micrographs of spinal cultures labeled by LY and GFAP. Bar plots summarize (F) LY-GFAP colocalization and (G) LY fluorescence intensity. * *P* < 0.033, ** *P* < 0.002, *** *P* < 0.001.

In GFAP-positive cells, pro-inflammatory CKs are reported to increase the permeability of membrane hemichannels (HC), a pathway for diffusion of small molecules or ions contributing, although not exclusively, to inflammation spread and to aberrant asynchronous calcium signaling among astrocytes in cultured spinal explants.^25^ We quantified Ca^2+^ oscillations synchrony by measuring the cross-correlation index (CI; see Experimental Section) between pairs of active astrocytes. In Figure 3A, superimposed fluorescence tracings are typical examples of simultaneously recorded pairs of glial cells (visualized by Fluo-4; Figure 3B) located at comparable distance in the four conditions (bar plot in Figure 3C). As summarized by the bar plot in Figure 3D, control and s-GO treated cells showed synchronized oscillations with similar CI values of 0.49 ± 0.06 in control (n = 8) and 0.69 ± 0.11 in s-GO (n = 6; *P* > 0.033). In CKs, glial cells displayed a statistically significant (*P* < 0.033) lower CI (0.22 ± 0.02 in CKs, n = 8); such a desynchronization was fully prevented by s-GO coapplication (0.64 ± 0.07, n = 6; CKs vs CKs+s-GO *P* < 0.002).

We used the low molecular-weight dye lucifer yellow (LY; 1 mM) to quantify HC permeability^47,48^ in astrocytes in all four conditions. In Figure 3E, representative micrographs show LY and GFAP colabeling, summarized in the plot in Figure 3F. CK treatment resulted in a statistically significant (*P* < 0.001) increase in double LY-GFAP positive cells when compared to control (11 ± 1 cells/field control and 31 ± 1 cells/field CKs; n = 9 fields each; Figure 3E,F). Such an increase was prevented in CKs+s-GO (12 ± 1 cells/field CKs+s-GO; CKs vs CKs+s-GO, *P* < 0.001), while s-GO per se did not differ from control (10 ± 1 cells/field s-GO, n = 9 fields each Figure 3E,F). Similarly, s-GO coapplied with CKs prevented the increase in LY fluorescence intensity summarized in Figure 3G (MFI 34 ± 7 au control, 108 ± 6 au CKs and 38 ± 4 au CKs+s-GO; CKs vs control and vs CKs+s-GO *P* < 0.001).

When preincubated in carbenoxolone (CBX, 200 μM, 10 min), an antagonist of gap-junction (GJ)^49,50^ and of active HCs,^51^ we detected a reduced dye uptake in all conditions, with no subsequent increase by CKs (Figure S2A,B). These results further supported that s-GO nanosheets modulated astrocyte reactivity to the inflammatory milieu in spinal slices, suggesting their specific ability to block HC increase in permeability.

### Quinine Activation of Connexin HC Induces Aberrant Calcium Signals Blocked by s-GO

**Figure 4:**
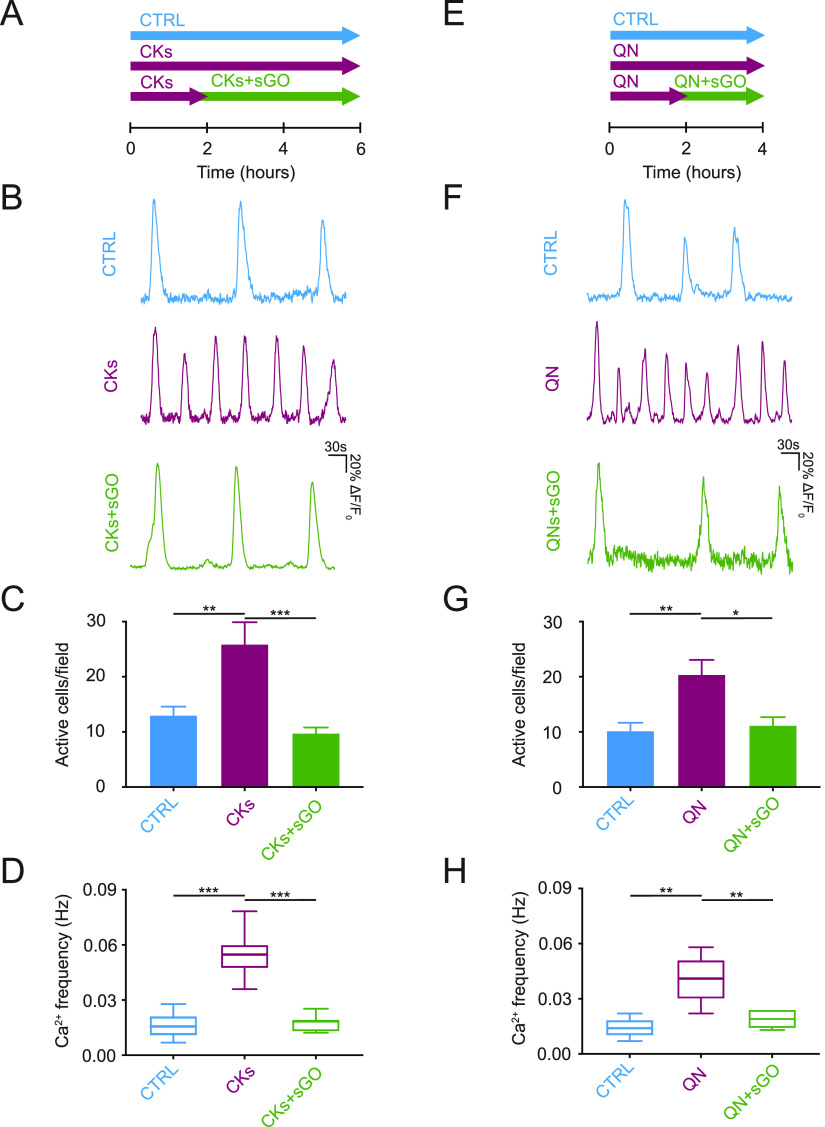
s-GO control of CKs induced or QN induced Ca^2+^ events increase in spinal cord astrocytes. (A) Schematic representation of the experimental design. (B) Calcium tracings in control, CKs, and CKs+sGO astrocytes. (C) Bar plot of number of active cells and (D) box plot of calcium events frequency. (E) Schematic representation of the experimental design. (F) Representative fluorescent traces depicting astrocytes calcium events in all conditions. (G) Bar plot of number of active cells and (H) box plot of calcium events frequency. * *P* < 0.033, ** *P* < 0.002, *** *P* < 0.001.

In the next set of experiments, s-GO was added after 2 h of inflammation induction by CK treatment (Figure 4A^27^) to challenge its ability to control aberrant Ca^2+^ signals and to exclude any direct interference of s-GO with CKs treatment such as adsorption on flakes surfaces. In Figure 4B, fluorescence tracings depict glial responses to CKs, in terms of increased number of active cells and oscillation frequency (bar plot and box-plot in Figure 4C and D, respectively). The delayed application of s-GO could still efficiently decrease the number of CK active glial cells (13 ± 1 cells/field control, 25 ± 4 cells/field CKs and 9 ± 1 cells/field CKs + s-GO treated ones, n = 8 each, *P* < 0.001; Figure 4C) and was able to tune back to control levels the oscillation frequency (0.016 ± 0.002 Hz control, 0.054 ± 0.004 Hz CKs and 0.017 ± 0.001 Hz CKs+s-GO, *P* < 0.001, Figure 4B–D).

We used quinine (QN; Figure 4E) to directly activate connexin HC.^47^Figure 4F–H show that QN (200 μM, 4 h) increased the number of active glial cells from 10 ± 1 cells/field (n = 5 control) to 20 ± 2 cells/field in QN (n = 5; *P* < 0.002, Figure 4G), as well as calcium oscillation frequency (from 0.014 ± 0.002 Hz in control to 0.040 ± 0.005 Hz in QN, *P* < 0.002; Figure 4F–H), mimicking aberrant calcium signaling brought about by CKs. QN-induced dysfunctional calcium signals were not detected when s-GO was coapplied after 2 h with QN (11 ± 1 cells/field and 0.019 ± 0.002 Hz QN+s-GO, n = 5; significance vs QN *P* < 0.033 and *P* < 0.002, respectively; Figure 4F–H). Thus, QN calcium dysregulation due to the opening of connexin HCs was blocked by s-GO, suggesting a direct impact on HC permeability. Such a hypothesis is further strengthened by the ability of s-GO to prevent the increase in double LY-GFAP positive cells following QN application (Figure S3A–C).

### s-GO Treated EAE Mice Show Milder Disease, Reduced Astrogliosis, and Increased Neuronal Survival

**Figure 5:**
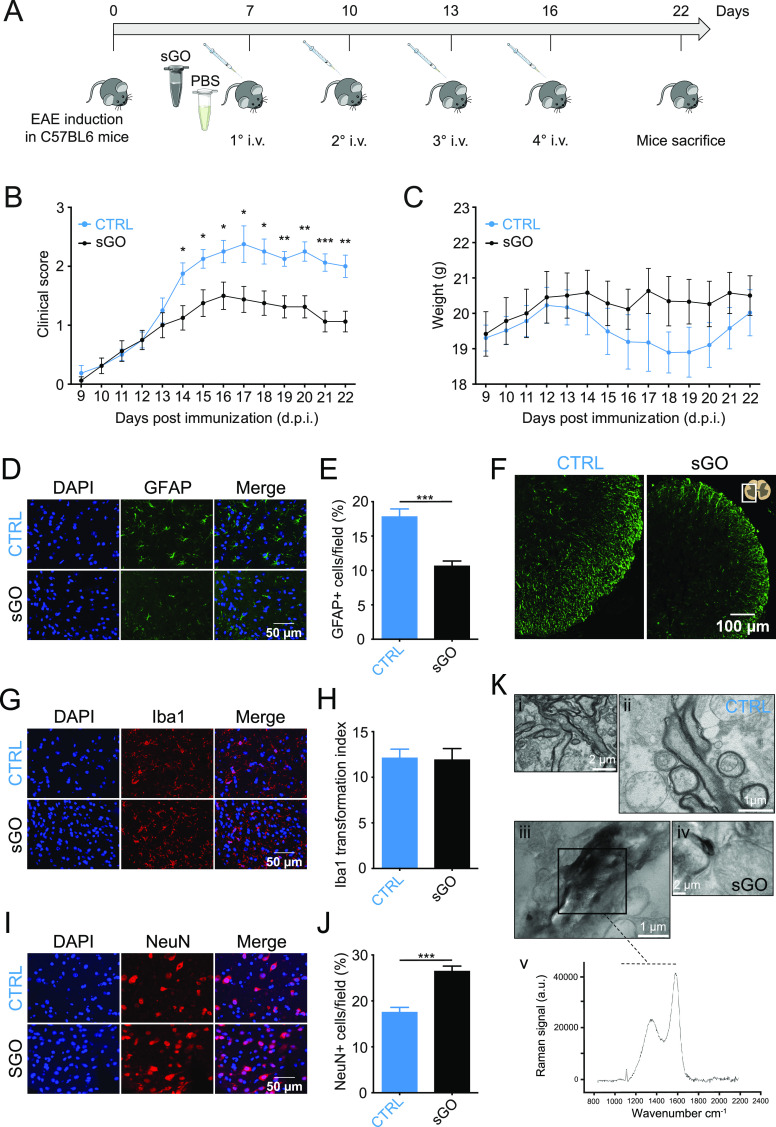
s-GO treatment in EAE mice ameliorates disease progression, reduces astrogliosis, and protects neuronal survival in the spinal gray matter. (A) At 7 days postimmunization (d.p.i.), EAE mice were treated by intravenous (i.v.) tail vein injections with 100 mL of PBS (control) or with s-GO (sGO) at 7, 10, 13, and 16 d.p.i. and sacrificed at 22 d.p.i. (B, C) Clinical score and weight (daily, 9–22 d.p.i.) of control and sGO EAE mice. (D) Fluorescence micrographs of spinal cord sections labeled with DAPI (in blue) and GFAP (in green). (E) Bar plot of the percentage of GFAP+ cells in control and in sGO EAE spinal gray matter. (F) Low magnification of EAE spinal sections in control and sGO EAE mice labeled by GFAP (in green). (G) Fluorescence micrographs of spinal cord sections labeled with DAPI (in blue) and Iba1 (in red). (H) Bar plot summarizes microglia Ti values in control and in sGO EAE spinal gray matter. (I) Fluorescence micrographs of spinal cord sections labeled with DAPI (in blue) and NeuN (in red). (J) Bar plot summarizes the percentage of NeuN+ cells in control and in sGO EAE spinal gray matter. (K) TEM micrographs of EAE mice spinal cord sections in (i, ii) control and (iii, iv) sGO; (v) box area: Raman spectrum in EAE sGO mice. * *P* < 0.033, ** *P* < 0.002, *** *P* < 0.001.

The capacity of s-GO nanosheets to modulate neuroinflammation in vivo was tested in EAE mice, which upon MOG_35–55_ immunization developed neuroinflammation-driven clinical and histological alterations, hallmarks of EAE (see Experimental Section(31)). After MOG_35–55_ administration, mice were treated with s-GO (4 mg/kg in 100 μL PBS; n = 8 animals) or PBS (100 μL, CTRL; n = 8 animals) through multiple tail vein injections at 7, 10, 13, and 16 days postimmunization (d.p.i., Figure 5A).

EAE onset and progression were monitored by daily evaluation of the clinical score (see Experimental Section) and animal weight for 3 weeks. Despite the comparable clinical values at 9 d.p.i. in CTRL and s-GO EAE mice (0.18 ± 0.13 CTRL and 0.06 ± 0.06 s-GO; *P* > 0.033; Figure 5B), in the following days CTRL animals showed a strong increase (worsening) of the clinical score, in agreement with the progressive development of the disease,^52^ while s-GO presented a significantly less severe progression (at 14 d.p.i. 1.8 ± 0.18 CTRL and 1.1 ± 0.2 s-GO, *P* < 0.033; at 22 d.p.i. 2 ± 0.1 CTRL and 1 ± 0.1 s-GO, *P* < 0.002; Figure 5B). The significantly lighter EAE disease progression in s-GO-treated animals was accompanied by a trend of more stable body weight, although not statistically significant when compared to CTRL progressive loss (Figure 5C).

To investigate the histological hallmarks of EAE, animals were sacrificed at 22 d.p.i. and we collected for analysis the spinal cord, the draining lymph nodes, and the spleen. In the spinal cord, we measured astrogliosis, microglial activation, and neuronal viability. In s-GO EAE spinal cord, gray matter GFAP positive cells were decreased (in %) when compared to CTRL EAE (17 ± 1% CTRL and 10 ± 1% s-GO, *P* < 0.001; Figure 5D,E), while no differences were found in white matter astrogliosis (Figure 5F; measured as corrected total cell fluorescence, CTCF, that was 4654074 ± 241754 au CTRL and 4081208 ± 243423 au s-GO; *P* > 0.033). Iba1-positive microglia Ti did not differ in the two EAE groups, with values indicative of ameboid reactive cells (Ti: 12 ± 1 CTRL and 11 ± 1 s-GO, *P* > 0.033; Figure 5G,H).

Neuronal viability, classically affected by neuroinflammation damages driven by EAE,^53^ was measured as % of viable neurons, identified by the specific neuronal nuclei marker NeuN. Figure 5I and J show an increase in NeuN+ neurons in s-GO treated vs CTRL (17 ± 1% CTRL and 26 ± 1% s-GO injected animals; *P* < 0.001). We additionally reported lymph node cell viability and phenotype as percentages of T, B, and dendritic cells (CD3+, CD19+, and CD11c+ cells, respectively). In comparing CTRL with s-GO EAE animals, we did not detect statistically significant differences in lymph node cell death (% propidium iodide, PI, positive cells: 11 ± 1% CTRL and 9 ± 1% s-GO, *P* > 0.033; Figure S4A) and in the phenotype of viable cells (by flow cytometry, for CD3+ cells 33 ± 2% CTRL and 28 ± 2% s-GO; for CD19+ cells 35 ± 3% CTRL and 34 ± 3% s-GO; for CD11c+ cells 1 ± 0.2% CTRL and 0.6 ± 0.1% for s-GO, all the P-values were > 0.033; Figure S4B). Figure S4C shows the proliferation responses of splenocytes and lymph node cells upon stimulation with MOG_35–55_ or with the specific mitogen phytohemagglutinin (PHA). In all cases, the exposure to s-GO did not impair the peripheral immune system cell viability or proportions between different phenotypes, and stimulated s-GO immune cells are even more prone to proliferate.

To explore whether s-GO reached the spinal cord in s-GO EAE, we used transmission electron microscopy (TEM; see Experimental Section). Such analysis revealed material deposits in s-GO EAE mice spinal cords (Figure 5K, panels iii and iv), while similar aggregates were never detected in CTRL EAE (Figure 5K, panels i and ii). Raman spectroscopy of such deposits confirmed a profile compatible with that of s-GO (Figure 5K, panel v). In agreement to that reported when multiple sclerosis animal models are treated with nanomaterials of comparable dimensions,^54^ we suggest that in EAE mice the compromised blood–brain barrier (BBB)^55,56^ allows s-GO nanosheets to pass the BBB and target the CNS, reducing astrogliosis and neuronal death in the spinal cord, without affecting the peripheral immune system (Figure S4).

Our evidence suggests that s-GO block the inflammation-induced calcium dynamics in in vitro astrocytes mainly by interfering with the activation of connexin HC.^25^ In addition, we demonstrate that the s-GO protective effect could be translated from cultured spinal explants to an in vivo model of EAE inflammatory disease.

The exposure of spinal explants to a CKs cocktail that mimics pro-inflammatory conditions associated with neurodegenerative disorders^57,58^ activates neuroinflammation onset and propagation, as shown by GFAP-positive astrocytes, Iba1-positive microglia, astrocyte-calcium, and synaptic network reactivity.^25−27^ These pathological aspects involving different cell types are reverted by s-GO treatment, indicating that the nanomaterial could target an upstream key component of the pro-inflammatory cascade. Reactive astrocytes in cultured slices are identified by the functional features of calcium dynamics (resistant to TTX, low pace kinetics, enhanced by CKs;^25^ or by HC activators^47^) and cell location (close to ventral neurons^25^). We focus on aberrant calcium activity, which mediates inflammatory cytokines and chemokines release in response to danger signals,^25^ as a hallmark of reactive astrocytes, and we suggest this as the main target of s-GO protective effects.

s-GO flakes were reported to interface exoendocytotic membrane dynamics at the nanoscale, interfering with shedding of membrane vesicles in astrocytes^20^ and with presynaptic vesicle release in neurons.^18^ These effects, potentially involved in tuning spinal circuit inflammatory reactivity, are unlikely in the current study, since both require prolonged exposure (days to weeks) to localized delivery of s-GO at high concentrations^18,20,59^ and, in spinal explants, involve the activation of microglia.^59^ Accordingly, in the current tests, in the absence of neuroinflammation, s-GO per se do not modulate neuroglia morphology, astrocyte calcium dynamics, or synaptic activity. We also exclude a direct interference, such as a mere adsorption of pro-inflammatory cues by s-GO flakes, since the nanomaterials are effective even when added 2 h after CKs exposure.

We can speculate that s-GO, which feature adhesion to complex patches of cellular membranes,^60^ specifically target HC permeability in reactive cells.

Nonjunctional HCs in astrocytes activate upon inflammation,^61,62^ allowing the pathological flow of ions and molecules, which promote neuroinflammation spreading.^8,63^ In our previous study, we reported that increased HC permeability was feeding aberrant calcium signaling and contributed to astrocyte inflammatory status in spinal explant slices.^25^ Here, pro-inflammatory HC activation is supported by CKs recruiting of active astrocytes with enhanced and desynchronized calcium oscillations^25^ together with the increased LY uptake in GFAP positive cells, an assay used to monitor HC permeability.^47,48^ LY uptake is significantly reduced by CBX, a HC inhibitor,^51^ strengthening the involvement of such proteins in reactive astrocytes calcium signaling pathways. Indeed, in the absence of the inflammatory milieu, the application of QN, a HC activator,^47^ induces changes in astrocyte calcium dynamics reminiscent of those provoked by neuroinflammation, efficiently blocked by s-GO. Although we cannot exclude the contribution of other membrane pathways to calcium microdomains, our results strengthen the link among HCs opening, calcium signals in reactive astrocytes, and inflammation,^64^ and we propose that s-GO protective effects can be mediated by a reduction in HC permeability, interrupting a vicious cycle in the spreading of neuroinflammation. We assess s-GO treatment of EAE mice, modeling multiple sclerosis, a well-known neuroinflammatory disease.^63^ In EAE mice, the disorder arose and progressed within few weeks, showing well-characterized patterns of histological and clinical aspects^65^ including astrogliosis and neuronal loss in the spinal cord-gray matter.^65^ s-GO delivery, without altering the function of the peripheral immune system, reached the spinal tissue and control astrocyte reactivity in the spinal cord gray matter, favoring neuronal survival. Such tissue protection is accompanied by an ameliorated clinical phenotype. To note, in EAE, due to the repeated s-GO administrations, we cannot rule out s-GO control over CNS synaptic activity,^18,21^ contributing to spinal neurons protection against neuroinflammation.^26^

Nanomaterials are increasingly engineered to treat neuro-pathologies including neuroinflammation.^66^ In preclinical animal models, such as EAE, such engineered nanoparticles were developed for targeted delivery of tolerogenic molecules to reduce human autoimmunity in an antigen-specific manner^67^ or more in general to favor therapeutic drug delivery based on nanotechnology approaches.^68^ For example, liposomes, polymeric nanoparticles, dendrimers, and dispersed carbon nanotubes were engineered to vectorize anti-inflammatory drugs and improve their pharmacokinetic properties; unfortunately, cell toxicity issues emerged when using many of these engineered nanomaterials.^69^ Differently, s-GO showed an intrinsic ability to modulate reactive cells, in the absence of cell toxicity, as also shown by γ-H2AX assay^70^ (Figure S5), excluding nuclear damage.

Although promising, currently the clinical translation of nanomaterial-based therapeutic approaches for the treatments of nervous system pathologies is limited by the lack of standard procedures for assessing the toxicology of these novel compounds.^71^ How long s-GO persist in the body and their potential translocation to secondary vital organs or nodules of the lymphatic system are all factors that should be considered in future clinical studies.

## Conclusions

Cell-targeting therapeutics in the CNS might take advantage of nanomaterial-based biointegrated platforms and, more in general, of engineered nanosized materials. In the current work, we exploit GO nanoflakes with small lateral size (s-GO) to target the over-reactivity of astrocyte, an early event linked to neuroinflammation spreading, and a shared hallmark of CNS disorders. s-GO flakes lessen astrocyte reactivity to inflammation by downregulating hemichannel opening and aberrant calcium signaling. We translated our in vitro findings to an in vivo mouse model of immune-mediated neuroinflammation disease, where s-GO reduced astrocyte reactivity and improved neuronal survival. This tissue protection was accompanied by an ameliorated clinical phenotype. The potential hold by s-GO to target astrocyte dysregulation in vivo controlling neuroinflammation neuronal damage might impact future strategies to treat CNS pathological conditions.

## Limitations of the Study

Multiple sclerosis is a human complex, multifocal disease that features distinct disabilities. Astrocytes regulate neuroinflammatory responses implicated in the development of EAE disease, an animal model of multiple sclerosis. In EAE, through active gliosis and upregulation of immune related genes^72,73^ astrocytes, in particular those located in the spinal cord, contribute to disease progression and may represent potential targets for therapeutic intervention.^1,10^ In our study, we characterize in vitro astrocyte reactivity when involved in neuroinflammation progression ignited by transient CKs exposure. Since we measured cell calcium responses and we monitored the emergence of gliosis in spinal cord explants, we propose a mechanism through which s-GO, by targeting specialized membrane channels, prevent dysregulated calcium signaling and the neuroinflammation cascade involving astrocytes. We further test s-GO in EAE mouse in vivo, in which repeated administrations of s-GO reduced astrogliosis and neuronal cell loss in the spinal cord, accompanied by ameliorated clinical signs.

Despite these encouraging results, our in vivo observations remain limited to a proof-of-concept experiment which might indicate novel targets, channel permeability in astrocytes, and unconventional tools, nanomaterials, to optimize neuroprotective treatment developments. Translational implications of s-GO are still in their infancy due to the lack of in-depth pharmacological investigations or of ad hoc studies addressing potential toxicity when using s-GO based vectorized systems in EAE. Dedicated pharmacokinetic and pharmacodynamic studies are warranted. Furthermore, the current study should be extended to other animal species and explore if there is any sex dependent effect.

In general, the biological tolerability and biocompatibility of s-GO injectable suspensions has been addressed in numerous studies performed previously using the same material in naïve, healthy animals. The rapid excretion, upon intravenous administration, through urine of a large fraction of the s-GO nanosheets^74^ and the absence of pathogenic alterations in the organs of accumulation,^75^ are suggestive of an overall biological profile for s-GO that consists of elimination and degradation by endogenous enzymes^76,77^ with no consequent adverse effects.

## Experimental Section

### Graphene Oxide Nanosheet Preparation

GO was obtained from graphite powder (Sigma-Aldrich, UK) and biology grade materials were derived as previously described (Hummers’ methods).^78^ We used non pyrogenic materials to guarantee endotoxin-free GO as previously described.^32,79^

### Atomic force microscopy

Atomic force microscope (Asylum MFP-3D, Oxford instruments), standard air-tapping mode (silicon probes Ted Pella, silicon probes), 300 kHZ resonance frequency, and 40 N/m force, was used. We used mica surface (Ted Pella, freshly cleaved) layered by 20 μL of poly-l-lysine 0.01% solution (Sigma-Aldrich), and, upon wash with water, 20 μL of GO suspension (100 μg/mL) was drop casted. Gwyddion software (http://gwyddion.net, version 2.56) was used to image processing. ImageJ software (https://imagej.nih.gov) with AFM images 5 μm × 5 μm height distribution analysis allowed was used to obtain the lateral dimensions values.

### Scanning Electron Microscopy

Magellan 400 L field emission scanning electron microscope (Oxford instruments; ICN2 Electron Microscopy Unit) was used with Everhart-Thornley (secondary electrons detector) at 20 kV (acceleration voltage) and 0.1 nA (beam current). In each sample, 20 μL of GO material (100 μg/mL) was deposited on an Ultrathin C on Lacey C grid. ImageJ software was allowed to obtain the lateral dimension distribution.

### Raman Spectroscopy

Confocal Raman microscope (Witec, at RT) with 632 nm laser excitation and 600 g/nm grating allowed us to acquire Raman spectra. Single Raman spectra were collected (from several spots, 1 mW and 10 s irradiation) using a 50× objective. Drop casted samples (20 μL) were analyzed by Origin software. I_D_/I_G_ intensity ratio was extrapolated by peak intensities with no baseline correction.

### Zeta Potential Measurements

Zeta-sizer Nano ZS (Malvern instruments) at the ICN2Molecular Spectroscopy and Optical Microscopy Facility was used to measure zeta potential (ζ). Each sample (20 μg/mL GO) was measured three times at room temperature with automatic refractive index and viscosity for water dispersant settings.

### Organotypic Spinal Cord Cultures and Treatments

All experiments were in accordance with the EU guidelines (2010/63/UE) and Italian law (Decree 26/14) and approved by the authority veterinary service and by our institution (SISSA) animal wellbeing committee (OBPA). Italian Ministry of Health (no. 22DABNQYA) animal use approval and EU Recommendation 2007/526/CE.

Organotypic spinal cord and dorsal root ganglia slices were obtained as previously described.^23,26,27^ Experiments of immunofluorescence, LY assay, calcium imaging, and patch clamp recordings were performed after 6 h incubation with the following treatments: (i) CTRL, standard medium; (ii) s-GO, standard medium and s-GO at 25 μg/mL (54); (iii) CKs, standard medium and a cocktail of the mouse recombinant cytokines (10 ng/mL each) TNF-α (R&D systems, #210-TA/CF), IL-1β (R&D systems, #M15330) and GM-CSF (R&D systems, #P04141);^26,27^ (iv) CKs+s-GO, standard medium, CKs cocktail, and s-GO at 25 μg/mL. In another set of experiments, s-GO were applied 2 h after the application of the CKs cocktail (delayed application), for a total time of 4 h coincubation. A similar time line was used in experiments with quinine (QN, 200 μM, Sigma), where sGO were applied for 2 h after QN application.

The experiments on the genotoxicity of s-GO were carried out exposing slices to s-GO at 25 μg/mL for 6 h. In the case of the excitotoxicity damage (positive control), another set of slices was treated with glutamate (100 μM) for 1 h, and then fresh medium was replaced for 24 h before immunofluorescence.

### Immunofluorescence Imaging and Analysis

Immunofluorescence staining was performed as previously reported.^26^ Secondary antibodies: Alexa 488 goat antimouse (1:500, Invitrogen), Alexa 594 goat antirabbit (1:500, Invitrogen). DAPI (Thermo Fisher Scientific) was also added to mark nuclei. We used Fluoromount-G (Invitrogen) to mount samples and Nikon A1R confocal microscope to acquire images. In the case of GFAP-Iba1 analysis, we acquired confocal sections every 1 μm with total Z-stack thickness 5 μm by 40× (0.95 NA) objective, while in the case of GFAP/γ-H2AX, we performed confocal section every 0.5 μm to a total Z-stack thickness of 15 μm by 100× (1.45 NA) objective. Confocal sections were acquired every 1 μm to a total Z-stack thickness of 5 μm. Analysis was performed using Fiji software on three independent culture series. Regarding the morphology analysis of microglia, we quantified the area and perimeter to compute the Ti,^26^ which is indicative for the microglia ramification status, while the astrocyte quantification of fluorescence intensity was performed by measuring the mean gray value. The analysis of H2AX phosphorylation at Ser 139 was carried out by counting the number γ-H2AX foci formation per nuclei colocalizing with GFAP and the number of γ-H2AX/DAPI/GFAP positive cells.

### Live Cell Ca^2+^ Imaging

Spinal organotypic slices were incubated in Fluo-4 AM (4 μM, Invitrogen; 1 h, 37 °C). After the loading and the de-esterification of the dye, the cultures were imaged by an inverted microscope (Nikon Eclipse Ti–U). At room temperature (RT), they were perfused continuously at 5 mL/min with a saline solution (in mM: 150 NaCl, 4 KCl, 1 MgCl_2_, 2 CaCl_2_, 10 HEPES, 10 glucose (pH = 7.4). Imaged with a 40× objective (PlanFluor, 0.60 NA), cultures loaded with the Ca^2+^ sensitive dye were excited with a mercury lamp at 488 nm. We used a 395 nm dichroic mirror and DN filter (1/32), and images were acquired at 7 fps every 150 ms by an ORCA-Flash4.0 V2 sCMOS camera (Hamamatsu) and a setup controlled by HCImage Live software. Initial recordings (10 min) in saline solution were made to analyze the spontaneous neuronal and glial activity prior to tetrodotoxin (TTX, 1 μM, Latoxan;^25^) application. Under these recording conditions, astrocyte activity was visualized^25^ in the premotor region of the slice ventral zone. The recorded images were processed identifying regions of interest (ROIs) around cell bodies with Fiji software. Related tracings were transferred to Clampfit software (10.6 version; Molecular Device LLC, US) and analyzed off-line to quantify the number of oscillating glial cells, the frequency of oscillations, their IEIs, and the CI. Ca^2+^ oscillations were measured as ΔF/F_0_, where ΔF is the fluorescence increase over the baseline, and F_0_ is the baseline level of fluorescence (as the median of the frame fluorescence values), calculated using the following formula:
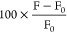
with F, fluorescence value and F_0_, baseline fluorescence.

### Luminex Assay and Analysis of Cytokines and Chemokines

In the supernatants obtained from organotypic slices, a panel of 13 CKs and chemokines was analyzed (IFNγ, IL1α, IL1β, IL4, IL6, IL10, IL12p40, IL12p70, IL17, C-X-C motif ligand 10 [CXCL10], C-C motif ligand 2 [CCL2], C-X-C motif ligand 2 [CXCL2], TNFα) by means of Milliplex assay (Merck Millipore, USA, #MCYTOMAG-70K-13), and Bio-Plex device (Bio-Rad, USA), as previously reported.^25^ CKs and chemokines amounts were expressed as pg/mL with a detection threshold of 1.0 pg/mL for all the analytes. Data were analyzed by calculating differences (Δ) between CTRL and CKs-stimulated organotypic cultures (ΔCKs – CTRL) or between organotypic cultures stimulated with both CKs and s-GO or only with s-GO (Δ [(CKs + s-GO) – s-GO]). Analysis was performed on three replicates for four organotypic cultures, corresponding to 12 measurements per group.

### Electrophysiological Experiments

Electrical activity of interneurons, visually identified in the ventral horn^80,81^ through an inverted microscope (Nikon Eclipse TE200), was recorded by using whole cell patch clamp technique. During experiments, slices were perfused with the supra-mentioned standard saline solution.

Patch pipettes (4–7 MΩ) were filled with (in mM) 120 K gluconate, 20 KCl, 10 HEPES, 10 EGTA, 2 MgCl_2_, 2 Na_2_ATP (pH = 7.3 with KOH and 295 mOsm of osmolarity). Recordings were performed at RT. We used a Multiclamp 700A amplifier (Axon Instruments), 10 kHz sampling, and signals were digitized with the pCLAMP software (Molecular Device LLC, US) for off-line analysis.

In voltage clamp mode, in order to avoid significant distortions of synaptic currents, we considered only recordings with series resistance <20 MΩ, not compensated for. Liquid junction potential was −14 mV, and values of potential were not corrected for it.

No differences were observed in membrane capacitance (43 ± 4 pF control, 40 ± 6 pF s-GO, 39 ± 4 pF CKs, and 44 ± 8 pF CKs+s-GO) and input membrane resistance (425 ± 106 MΩ control, 472 ± 86 MΩ s-GO treated, 263 ± 18 MΩ CKs, and 515 ± 120 MΩ CKs+s-GO) of spinal neurons recorded upon various treatments.

Spontaneous activity was monitored at a holding potential of −56 mV. sPSCs analysis was performed using the Clampfit 10.6 software (Molecular Device LLC, US). For each recording, ≥ 100 sPSCs were collected, and the inter-event intervals and peak amplitudes were measured.

### Lucifer Yellow (LY) Uptake

For dye uptake experiments, we used LY (Sigma). Cultures were incubated (10 min) at 37 °C with 1 mM of LY in standard physiological solution and washed three times with the same solution without LY. To investigate how GJs and HCs contributed to dye uptake, an independent set of spinal cord slices was previously treated with CBX (Sigma) at 200 μM for 10 min and later incubated with LY. Cultures were fixed with 4% PFA, and subsequently astrocytes were labeled using anti-GFAP as described earlier. Images were acquired at the confocal microscope using 20× and 40× objectives, with a step of 1 μm for a total Z-stack 5 μm thickness. Analyses of LY-GFAP double positive astrocytes and the quantification of LY fluorescence intensity were performed using Fiji software on three independent culture series.

### Induction of EAE

Experiments were carried out on 6–8 weeks old C57BL/6 female mice (Charles River Laboratories, Italy). Animals (CeSaL at University of Florence, Italy) were kept at a temperature of 23 °C, 12 h (7am–7 pm) light/dark cycle. Two experimental series were performed for a total of 16 mice. All experiments were authorized according to the Committee for Animal Care guidelines (d.lgs. 26/2014; authorization n. 1145/2020-PR).

Animals were immunized through three subcutaneous (s.c.) injections (two interscapular and one at the base of tail) of 200 μg of MOG_35–55_ peptide (MEVGWYRSPFSRVVHLYRNGK, purity of 85%; Espikem, Italy) solved in sterile water, emulsified with an equal volume of Complete Freund’s Adjuvant (CFA), and supplemented with 7 mg/mL of *Mycobacterium tuberculosis* (H37Ra strain; NR Nannini, Italy). To increase the BBB permeability, each mouse received, the day of immunization and after 48 h, 500 ng (in a volume of 100 μL) of *Bordetella pertussis* toxin (PTX; Merck, Germany) dissolved in PBS by intraperitoneal (i.p.) injection. All the experiments with animals were blinded. EAE mice were monitored daily for weight and signs of clinical disease, with (0) no signs of disease; (1) hind limb or limp tail weakness; (2) hind limb and limp tail weakness; (3) partial paralysis of hind limb; (4) complete paralysis of hind limb; or (5) moribund or death by EAE. If reaching the score 4, mice were sacrificed for ethical reasons.

### In Vivo Treatment with s-GO

Mice were separated in two groups: one treated with PBS as control, and one treated with s-GO. The attribution of animals to the experimental groups was randomized. s-GO were administered four times (7, 10, 13, and 16 days postimmunization, d.p.i.) by intravenous (i.v.) injection in the tail vein, at 4 mg/kg of concentration in a PBS volume of 100 mL for injection. The dose used for the pilot study in vivo was estimated based on our in vitro results (10–25 μg/mL^18,21^) and on our previous in vivo results with local s-GO tissue administration (to the hippocampus 50 μg/mL and to the amygdala 50 μg/mL^18,21^). In parallel, an equal volume of PBS was administered to control mice. At 22 d.p.i., after mice sacrifice draining lymph nodes, spleen and spinal cord were taken for cytological and histopathological analyses.

### Histopathological Evaluation on Mice Spinal Cord

After removal, mice spinal cord was immediately fixed by 4% PFA, paraffin embedded and microtome-sectioned in 5 μm slices. Spinal cord sections were treated to remove paraffin by two steps of 5 min each in xylene and descending alcohols (100%, 95%, 70%, and 50%, 3 min each) until distilled water, then destined to immunofluorescence analysis. Sections were treated for antigen retrieval with buffer-citrate (citric acid 10 mM with pH = 6, Tween 0.05%; Sigma, USA) at 95 °C for 20 min, then cooled, washed in PBS thrice, and permeabilized by FBS 5% and Triton 0.3% in PBS, 30 min at RT. Slides were then washed thrice with PBS and labeled at 4 °C overnight with primary monoclonal antibody rat antimouse GFAP (clone 2.2B10), to detect astrocytes, with polyclonal antibody rabbit antimouse Iba1, to detect microglia or with polyclonal antibody NeuN to label neuronal nuclei. Afterward, sections were PBS washed thrice and labeled 2 h, RT, in the dark, with appropriate secondary antibodies, including Alexa Fluor 488 goat antirat IgG (H+L) or Alexa Fluor 594 donkey antirabbit IgG (H+L), both from Thermo Fisher Scientific, USA. All antibodies (Thermo Fisher Scientific, USA) were used 1:500 diluted in permeabilizing solution. After secondary antibody, spinal cord sections were PBS washed three times and mounted using mounting medium containing DAPI to detect cell nuclei (Prolong Gold with DAPI, Life Technologies, USA).

### Analysis of Neurons, Astrogliosis, and Microglial Activation

Astrogliosis in spinal cord gray matter and neuronal state were evaluated by manual count of GFAP+ (astrocytes) or NeuN+ (neurons) using ImageJ software (NIH, USA) cells in slide fields at 40× magnification. For each mice group, a range of 5–10 random fields per slide was evaluated (each slide field is equal to an area of approximately 0.04 mm^2^). Astrogliosis in white matter was analyzed by ImageJ software by calculating the CTCF, measured as integrated density – (area of a selected cell × mean fluorescence of background readings).

On Iba1-labeled spinal cord sections, microglial activation was analyzed on selected areas from a range of 5–10 random fields/slide using ImageJ software, evaluating the morphology variation of microglia, to measure the Ti, as previously described in this manuscript.

### Cytological Analyses: Lymph Node Cells Viability and Phenotype

Draining lymph nodes of mice were washed with 1% of penicillin/streptomycin and 1% fungizone in PBS and mechanically dissociated on sterile Falcon 70 μm cell strainer. Lymph node cells were centrifuged 1300 rpm, 10 min, and counted. Cells of lymph nodes were analyzed by CyFlow Space flow cytometer (Sysmex Partec, Gemany) for viability using propidium iodide (PI; 2.5 μg/mL; Molecular Probes, USA) to detect necrotic cells, and for phenotype by labeling cells for 20 min, at RT, with the following antimouse antibodies: CD3 FITC (clone 145–2C11, BioLegend, USA), CD19 PE (Miltenyi Biotec, Germany), CD11c PE (clone N418, eBioscience, USA). Flow cytometry data were acquired following standard guidelines^82^ and analyzed by the FloMax software (Sysmex Partec, Germany). CD3+, CD19+, and CD11c+ were gated on a forward scatter (FSC) vs a side scatter (SSC) dot plot. Gates were labeled according to FloMax software default (Figure S2B; gate “R1” includes the whole reference cell population; gate “Q4” includes CD3+, CD19+, or CD11c+ cells within the “R1” gate, whereas “Q3” includes cells negative for all the above-mentioned markers).

### Statistical Analysis

All the data sets underwent to the normality analysis to assess if data were as in a Gaussian distribution. On this basis, concerning the work on organotypic spinal cord slices, the data sets were processed with ordinary one-way ANOVA test to measure if differences were statistically significant (post hoc test by Holm-Sidak comparisons), otherwise Kruskal–Wallis test was used (post hoc test by Dunn’s multiple comparison). Cumulative probability analyses were performed using the Kolmogorov–Smirnov test among groups. Regarding in vivo experiments, the progression of clinical score and weight were evaluated by using two-way ANOVA test to measure significant differences among groups, with post hoc test Fisher’s LSD. Statistical significance of all the other results were tested as unpaired t Test (parametric data) or Mann–Whitney (nonparametric data). All the data are expressed as Mean ± SEM, n = number of cultures, unless stated otherwise.

## Supporting Information Available

Cytokines and chemokines measurements; LY uptake; s-GO effect on peripheral immune system; genotoxicity evaluation of s-GO impact; supplementary methods for UV–vis spectroscopy, X-ray photoemission spectroscopy, TEM analysis on mice spinal cord, Raman spectroscopy analysis; lymph node cells and splenocytes proliferation (PDF)

## Abbreviations

CKs: cytokines;
GO: graphene oxide
CNS: central nervous system
HC: hemichannel
s-GO: small graphene oxide
LY: lucifer yellow
CBX: carbenoxolone
GJ: gap-junction
CI: cross-correlation index
QN: quinine
