# Supplementary material for: Graphene Oxide Nanosheets Reduce Astrocyte Reactivity to Inflammation and Ameliorate Experimental Autoimmune Encephalomyelitis
Source: ACS Nano. 2023 Jan 24;17(3):1965–78. doi: 10.1021/acsnano.2c06609 (PMC9933621; doi:10.1021/acsnano.2c06609)
Supplement: Supplementary file 1 — nn2c06609_si_001.pdf [file nn2c06609_si_001.pdf]

Graphene oxide nanosheets reduce astrocyte reactivity to inflammation and ameliorate experimental autoimmune encephalomyelitis

Giuseppe Di Mauro[1#], Roberta Amoriello[1,2#], Neus Lozano[3], Alberto Carnasciali[2], Daniele Guasti[2], Maurizio Becucci[4], Giada Cellot[1*], Kostas Kostarelos[3,5], Clara Ballerini[2*], Laura Ballerini[1*]

[1] *International School for Advanced Studies (SISSA/ISAS), 34136 Trieste, Italy*
[2] *Dipartimento di Medicina Sperimentale e Clinica, University of Florence, 50139 Florence, Italy*
[3] *Catalan Institute of Nanoscience and Nanotechnology (ICN2), 08193, Barcelona, Spain*
[4] *Dipartimento di Chimica "Ugo Schiff", DICUS, University of Florence, 50139 Florence, Italy*
[5] *National Graphene Institute and Faculty of Biology, Medicine & Health, The University of Manchester, Manchester, M13 9PL, United Kingdom*

[#] Equal contribution

*Correspondence to clara.ballerini@unifi.it cellot@sissa.it laura.ballerini@sissa.it

Supplementary information

Supplementary Figures

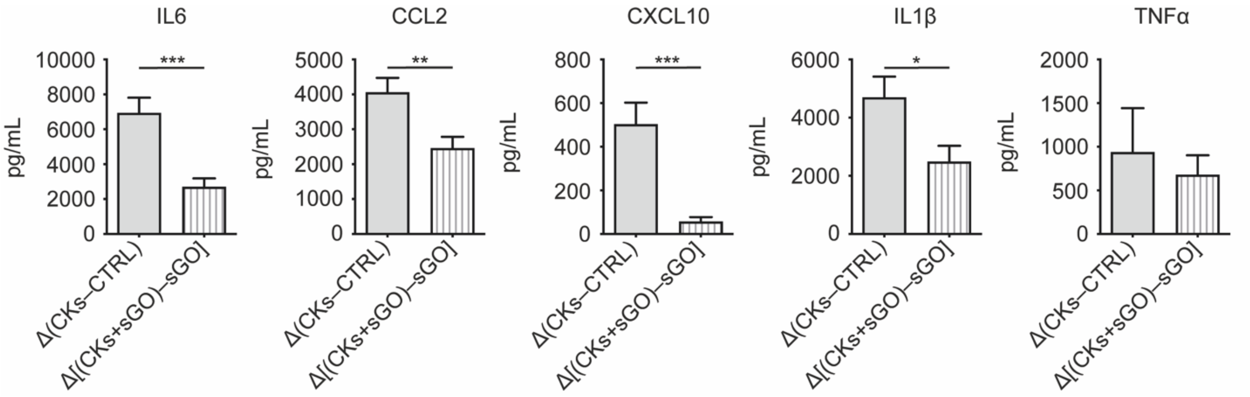

Figure S1 | Cytokines and chemokines measurements by Luminex assay in organotypic culture supernatants in CKs or CK+s-GO treatments. Concentration variations for IL6, CCL2, CXCL10, IL1β and TNFα, measured in organotypic culture supernatants by Luminex assay upon stimulation with s-GO alone, CKs, CKs + s-GO, or in unstimulated CTRL cultures. Values are reported as difference (Δ) calculated between CTRL and CK (Δ CKs-CTRL) and between (CKs + s-GO) and s-GO alone (Δ [(CKs + s-GO) – sGO]). Bar plots show a statistically significant reduction for IL6 (Δ (CKs-CTRL) = 6926 ± 883 pg/mL; Δ [(CKs+s-GO) - s-GO] = 2695 ± 496 pg/mL), CCL2 (Δ (CKs-CTRL) = 4058 ± 416 pg/mL; Δ [(CKs+s-GO) - sGO] = 2455 ± 329 pg/mL), CXCL10 (Δ (CKs-CTRL) = 502.4 ± 100 pg/mL; Δ [(CKs+s-GO) - s-GO] = 57.12 ± 20 pg/mL), and IL1β (Δ (CKs-CTRL) = 4696 ± 716 pg/mL; Δ [(CKs+s-GO) - s-GO] = 2486 ± 545 pg/mL). Although not statistically relevant, a similar trend was observed for TNFα (Δ (CKs-CTRL) = 938 ± 505 pg/mL; Δ [(CKs+s-GO) - s-GO] = 677.2 ± 226 pg/mL).

*P<0.001

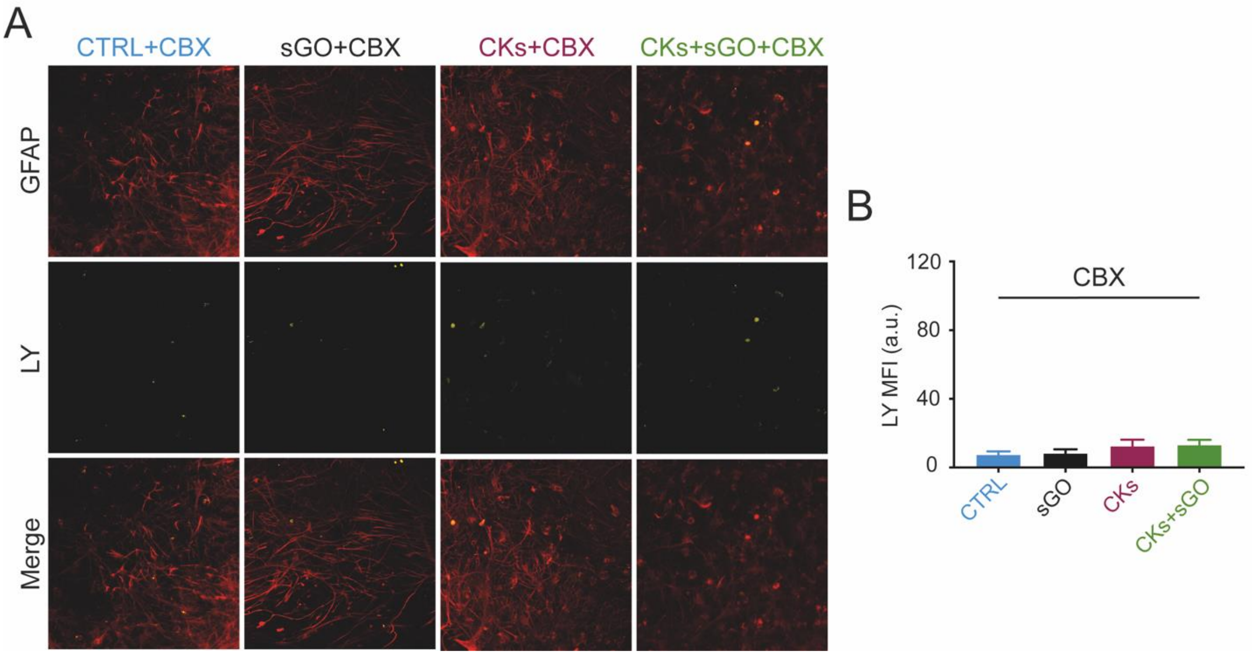

Figure S2 | LY uptake in spinal culture GFAP positive cells in the presence of CBX. Fluorescence micrographs of spinal cultures: live-uptake of LY and GFAP labeling in control, s-GO, CKs and CKs+sGO all in the presence of CBX (A). Bar plot summarizes LY fluorescence intensity in GFAP positive cells (B).

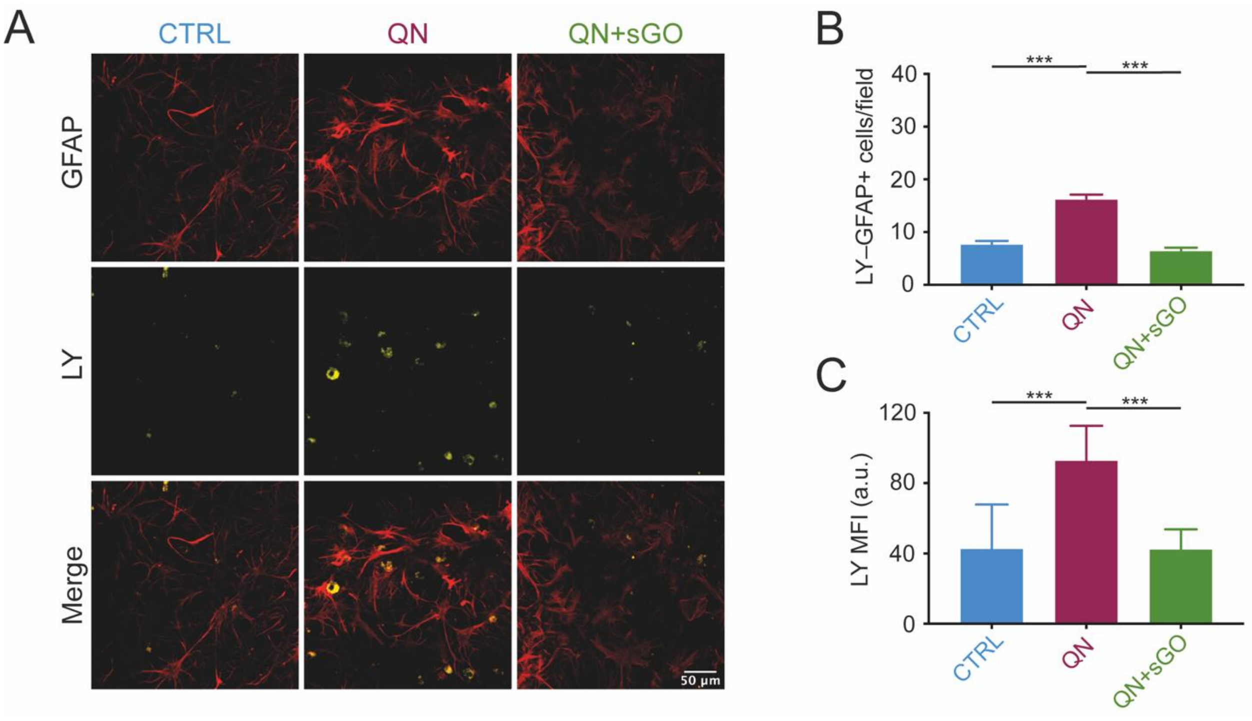

Figure S3 | LY uptake in spinal culture GFAP positive cells exposed to QN.

Fluorescence micrographs of spinal cultures: live-uptake of LY and GFAP labeling in control, QX exposed and QX exposed, but post co-incubated with s-GO (A). Bar plots summarize the number of LY and GFAP co-labeled cells/field (B) and LY fluorescence intensity in GFAP positive cells (C). * P<0.002

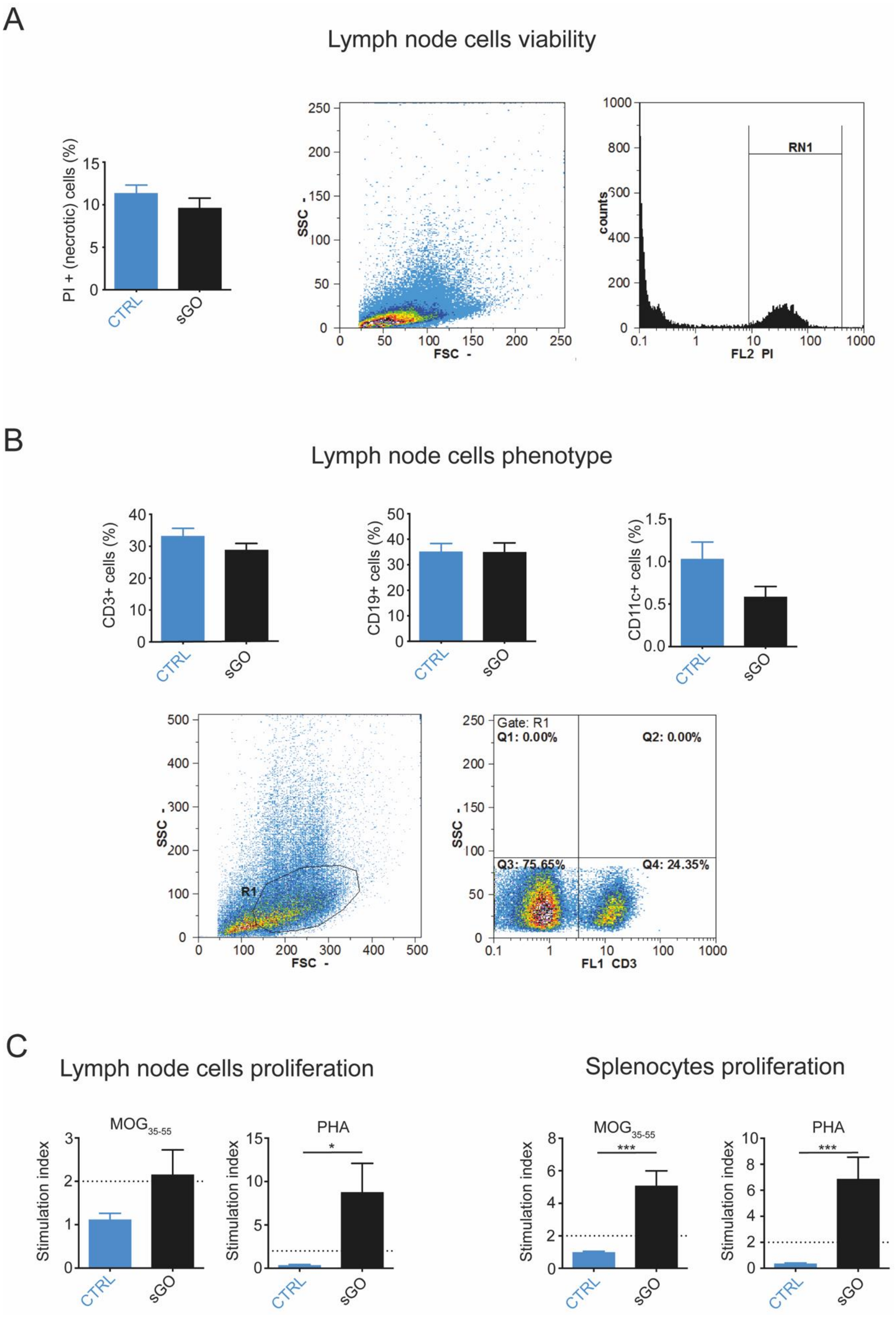
A
Lymph node cells viability
PI + (necrotic) cells (%)
CTRL
sGO
SSC -
FSC -
counts
RN1
FL2 PI
B
Lymph node cells phenotype
CD3+ cells (%)
CD19+ cells (%)
CD11c+ cells (%)
CTRL
sGO
R1
Gate: R1
Q1: 0.00%
Q2: 0.00%
Q3: 75.65%
Q4: 24.35%
SSC -
FSC -
FL1 CD3
C
Lymph node cells proliferation
Splenocytes proliferation
MOG35-55
PHA
Stimulation index
*
***
***
CTRL
sGO

Figure S4 | s-GO do not affect the peripheral immune system: cytological analysis of lymph node and spleen cells. Necrotic lymph node cells are reported (left) as percentage of PI positive cells, measured by flow cytometry and evaluated on the whole lymph node cells population; gating strategy is reported on the right (SSC: side scatter; FSC: forward scatter; gate “RN1”: PI+ cells) (A). Percentage of CD3+, CD19+ and CD11c+ cells of lymph nodes evaluated by flow cytometry. Gating strategy is shown in the lower dot plot panel (gate “R1”: lymphocytes; gate “Q3”: CD3- cells; gate “Q4”: CD3+ cells within the R1-selected cell population) (B). Proliferation of lymph node cells (left) or splenocytes (right) evaluated by 3H-thymidine incorporation assay (reported as stimulation index; SI). Cells were stimulated with MOG35-55 (50 µg/mL) or PHA (5 µg/mL) and considered proliferative when SI > 2.(C) * P<0.001.

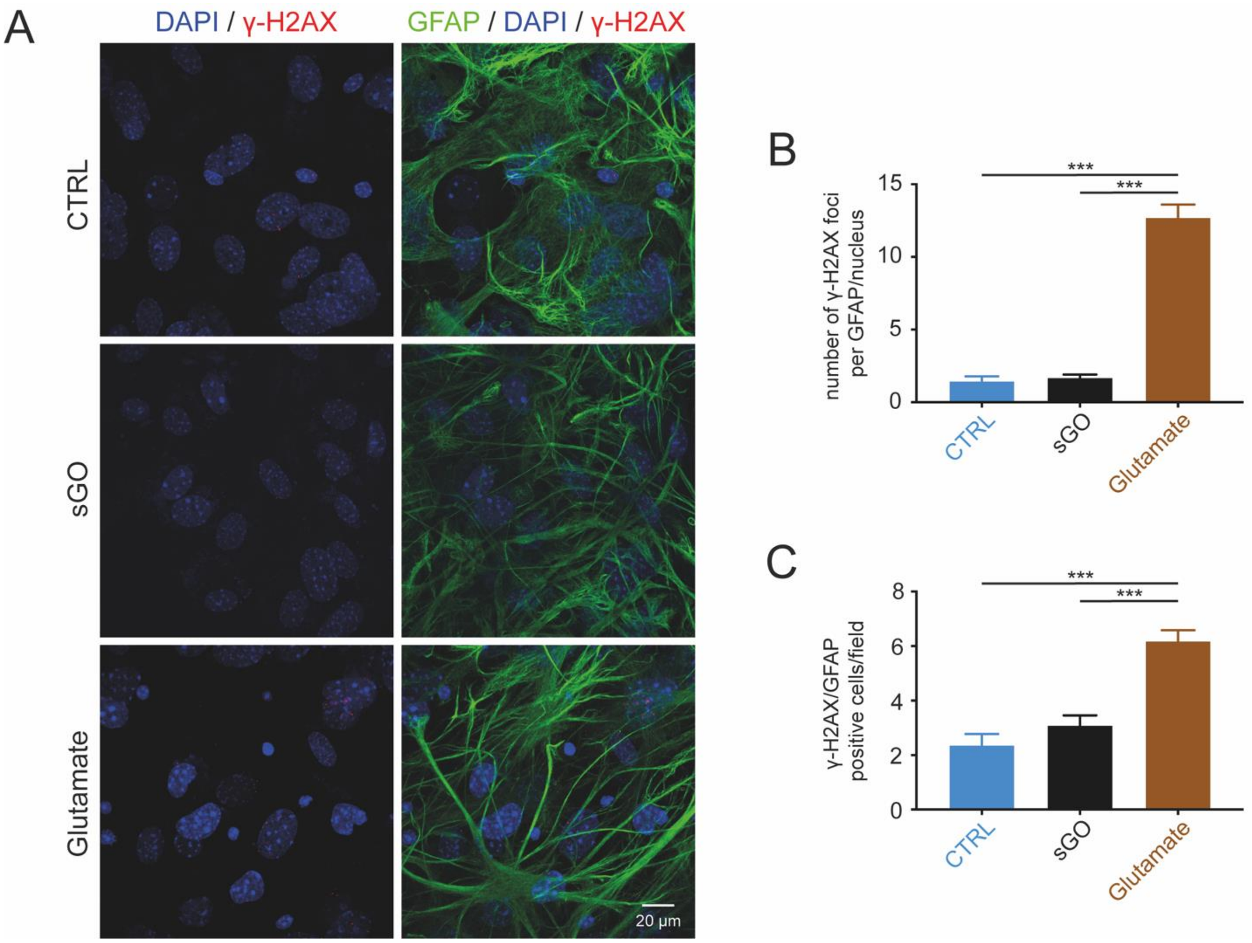

Figure S5 | Genotoxicity evaluation of s-GO impact on DNA double-strand breaks by measuring Histone H2AX phosphorylation at Ser 139 (γ-H2AX) reveals the lack of astrocytic nuclear DNA damage. Representative confocal images of γ-H2AX foci colocalizing with DAPI and GFAP labelling. For the experiments were used non-treated spinal cultures (CTRL), s-GO treated ones and, as additional positive control, nuclear damage was induced by exposing the cultures to an excitotoxic damage (Glutamate, 100 µM for 1 hour) (A). Bar plot summarizes the number of γ-H2AX foci/ nucleus of glial cell. No statistically significant differences were detected between CTRL and s-GO (γ-H2AX foci/nucleus of glial cell were 1.41 ± 0.33 in control vs 1.68 ± 0.22 in s-GO, n=11 for each condition), while cultures undergone to glutamate treatment reported a

significant increase in foci formation (12.70 ± 0.90, n=13, $P<0.001$ for both CTRL vs Glutamate and s-GO vs Glutamate) (B). Bar plot of the number of positive cells. No differences were detected between CTRL and s-GO treatments (2.36 ± 0.41 cells in CTRL vs 3.09 ± 0.36 in s-GO, n=11 for each condition), while glutamate treated samples showed a statistically significant increment in the number of cells positive to γ-H2AX (6.18 ± 0.40 cells, n=13, $P<0.001$ for both comparison: CTRL vs Glutamate and s-GO vs Glutamate) (C).

Supplementary methods

UV-Vis spectroscopy. Absorbance was evaluated by using a Nanodrop 2000c spectrophotometer (Thermo Scientific) at room temperature using a Hellma QS Quartz micro cuvette. GO samples were prepared in water in a concentration range of 2.5-20 μg/mL.

X-ray photoemission spectroscopy. XPS data was obtained using a Phoibos 150 (SPECS, GmbH) electron spectrometer equipped with a hemispherical analyzer, operating under ultrahigh-vacuum conditions, and with an Al Kα (hν = 1486.74 eV) X-ray source, at the ICN2 Photoemission Spectroscopy Facility. Charge effects on the samples were removed by taking the C1s line from adventitious carbon at 284.6 eV. Samples were prepared by deposition of 20 μg of GO material onto 5x5 silicon wafers (Ted Pella) and dried overnight. In order to estimate the photoelectron peak intensities CasaXPS software (http://www.casaxps.com) was used.

TEM analysis on mice spinal cord. Immediately after sacrifice, spinal cord from EAE s-GO- or PBS-treated mice was fixed overnight in Karnovsky solution at 4 °C, then treated in Millonig buffer containing 1% osmium tetroxide. Samples were dehydrated with increasing concentrations of acetone, therefore embedded in epon 812 Luft resin and sectioned with an ultramicrotome III LKB in 50-60 nm slices on a 300 mesh copper grid. Spinal cord samples were then stained with Uranyless TEM staining solution (Microtonano, Netherlands) and concentrated bismuth and observed with Jeol Jem 1010 microscope (Jeol, Japan, [1]).

Raman spectroscopy analysis. A portion of mice spinal cord was observed by Raman spectroscopy to confirm TEM detection of s-GO within the tissue. Raman spectra were obtained by a Renishaw RM2000 Raman microscope (Renishaw, UK) and the 514.4 nm line of an Ar^+ laser (model 163-M42-010 by Spectra Physics, USA), focused by an extra-long working distance Nikon M Plan achromatic objective capable of 60× (0.70 NA). Spatial resolution was about 1 μm. Power at the sample was up to 1.5 mW. The spectral resolution was 5 cm^{-1} (equivalent to 3 pixels on the CCD camera) determined from linewidth measurements on the first-order Raman band of bulk Silicon at 520 cm^{-1} [2]. The experimental conditions were the following: 514.5 nm excitation wavelength, laser power at the sample 0.4-1.5 mW, average of 5 spectra with 10-100 s integration time; spectra were recorded in the 850-2200 cm^{-1} Raman shift spectral range. Raman spectra were elaborated by the Microcal Origin software (Origin LabCorporation, USA) for data averaging and for baseline subtraction.

Lymph node cells and splenocytes proliferation. Mice spleen and draining lymph nodes were dissociated. Isolation of mononuclear cells (MNCs) from spleens was performed by density gradient centrifugation using Pancoll (with a density of 1.077 g/mL; Bioclass S.R.L., Italy) at 1500 rpm for 30 mins. Spleen MNCs were washed thrice and counted. Lymph node cells and splenic MNCs were then cultured in 96-well plates (Jet Biofil, China) at a density of 2x105 cells/well, in quadruplicate, in complete RPMI medium (5% fetal bovine serum, 1% penicillin/streptomycin, 1% pyruvate sodium, 1% L-glutammine and 1% Hepes buffer) and in three conditions: 1) unstimulated; 2) stimulated with 50 μg/mLof MOG35–55 or 3) stimulated with 5 μg/mL of PHA for 72 hours at 37°C and in 5% of CO2. 3H-thymidine at a concentration of 1 μCi/well (Perkin Elmer, USA) was pulsed for 8 hours, then cells were harvested by means of a Tomtec Harvester Mach III Cell Harvester (TomTec, USA) on a glass fiber filters. The proliferative response, measured as counts per minute (cpm) with a scintillation Trimux Microbeta 1450 counter (Wallac, USA), was valued as positive with a stimulation index (SI)>2 (SI = cpmstimulated cells/cpmunstimulated cells).

References

(1) Aldinucci, A.; Turco, A.; Biagioli, T.; Toma, F. M.; Bani, D.; Guasti, D.; Manuelli, C.; Rizzetto, L.; Cavalieri, D.; Massacesi, L.; Mello, T.; Scaini, D.; Bianco, A.; Ballerini, L.; Prato, M.; Ballerini, C. Carbon Nanotube Scaffolds Instruct Human Dendritic Cells: Modulating Immune Responses by Contacts at the Nanoscale. Nano Lett. 2013, 13 (12), 6098−6105 doi.org/10.1021/nl403396e.

(2) Richter, H.; Wang, Z. P.; Ley, L. The One Phonon Raman Spectrum in Microcrystalline Silicon. Solid State Commun. 1981, 39 (5). 625−629 doi.org/10.1016/0038−1098(81)90337−9.
